# Effect of Crystallinity on Young’s Modulus of Porous Materials Composed of Polyethylene Terephthalate Fibers in the Presence of Carbon Dioxide

**DOI:** 10.3390/polym14183724

**Published:** 2022-09-06

**Authors:** Takafumi Aizawa

**Affiliations:** Research Institute for Chemical Process Technology, National Institute of Advanced Industrial Science and Technology, 4-2-1 Nigatake, Miyagino-ku, Sendai 983-8551, Japan; t.aizawa@aist.go.jp; Tel.: +81-22-237-5211

**Keywords:** Young’s modulus, crystallinity, porosity

## Abstract

Carbon dioxide (CO_2_)-assisted polymer compression method is used for plasticizing polymers with subcritical CO_2_ and then crimping the polymer fibers. Given that this method is based on crimping after plasticization by CO_2_, it is very important to know the degree of plasticization. In this study, heat treatment was gently applied on raw material fibers to obtain fibers with different degrees of crystallinity without changing the shape of the fibers. Simultaneously, two types of sheets were placed in a pressure vessel to compare the degree of compression and the degree of hardness. Furthermore, a model was used to derive the relative Young’s modulus of porous materials composed of polymer fibers with different degrees of crystallinity. In the model, the amount of strain was calculated according to the Young’s modulus as a function of porosity and reflected in compression. Young’s modulus of porous polymers in the presence of CO_2_ has been shown to vary significantly with slight differences in crystallinity, indicating that extremely low crystallinity is significant for plasticizing the polymer by CO_2_.

## 1. Introduction

CO_2_ is a gas with low toxicity and is being used in the food [1] and pharmaceutical industries [2]. CO_2_ is known to specifically dissolve in polymers, and research on the mixture of polymers and CO_2_ has been actively conducted in relation to the polymer process [3] and polymer synthesis [4]. The dissolution of CO_2_ in solid [5] or melted polymers [6] has been used in the foaming process. The rapid expansion of supercritical solution [7], particles from gas saturated solutions [8], and supercritical antisolvent methods [9] have been proposed as processes that use CO_2_ to atomize the polymer.

Under these circumstances, the CO_2_-assisted polymer compression (CAPC) method, which is a method used to produce porous material by plasticizing and crimping polymer fibers with CO_2_ under subcritical conditions of vapor pressure at room temperature, was developed [10]. The process leads to the creation of porous materials with through holes in a short time. Until now, the CAPC method has been focused on properties and process conditions for practical use, such as porosity and pore diameter [11], adhesion strength [12], controlled release [13], air permeability [14], and mass production [15]. Recently, utility components, such as multilayer filters (considerations with classical model [16], considerations with deep-learning model [17]), application to bioplastics [18], and creation of reaction cartridges loaded with enzymes [19], have been developed. The CAPC method is used at room temperature. The method has the limitation that the polymers need to be plasticized under subcritical conditions, which are gentler than supercritical conditions. In other words, to understand the applicability of the CAPC method, one needs to understand the plasticization of the polymers involved. CO_2_ is known to easily dissolve into polymers, especially when dissolved in the amorphous part, by numerous viewpoints (reaction engineering of polymers in supercritical CO_2_ [20], model calculations of CO_2_ solubility [21], determination of CO_2_ solubility [22], and interaction between polymers and CO_2_ [23]). The degree of crystallinity of the polymers greatly affects the formation of foams by influencing the solubility and diffusion of CO_2_ [24]. In the case of semicrystalline polymers, the crystalline state of the polymer affects the nucleation of foam [25], and several approaches to strategically use such polymers to create foams have also been attempted [26]. Thus, there are many cases where solid polymers and CO_2_ processes require the consideration of crystallinity and crystalline state.

The crimping of fibers by the CAPC method does not occur in the absence of CO_2_. As a result, the plasticization of the polymer by CO_2_ is known to be essential. From previous studies, the plasticization of polymers by CO_2_ would be affected by the degree of crystallinity of the polymer. However, the effect must be evaluated using the measurement of Young’s modulus under high pressure, and there has only been a little research conducted on this topic. In the case of fibrous porous polymers, such as in the CAPC method, unless the fiber diameters are matched, it is impossible to observe only the effect of crystallinity, and unless the resin is made from the same pellets, it is not easy to observe only the effect of crystallinity because the resin may be affected by some other components, such as molecular weight distribution.

In this study, these problems have been solved by cold crystallization of polyethylene terephthalate (PET) nonwoven sheets by heat treating them for a given period above the glass transition point without changing the shape of the fibers and by adjusting samples with different degrees of crystallinity. Furthermore, analysis of Young’s modulus, both large and small, was carried out by piling up raw material sheets with different crystallinity values and placing them in a container to analyze how they crumpled after compression. The Young’s modulus for the porosity of the samples with different crystallinity was successfully obtained by analyzing the compression process with a model.

## 2. Materials and Methods

### 2.1. Materials

Nonwoven fabrics (average fiber diameter: 8 µm, basis weight: 30 g/m^2^) made by the melt blowing method [27] at Nippon Nozzle Co., Ltd. (Kobe, Japan) using PET pellets of TK3 (density: 1.34 g/mL) from Bell Polyester Products, Inc. (Houfu, Japan) were used as raw material fibers. The untreated samples were used as they were, while the samples with increased crystallinity were created by heat treating at 94 °C for 1.5 or 3 h to increase crystallinity without changing the fiber shape by gentle cold crystallization. The degree of crystallinity was checked by differential scanning calorimetry (DSC) on Thermo Plus Evo DSC 8230 (Rigaku Corporation, Akishima, Japan) under DSC measurement conditions of 9.39–10.40 mg sample weight and a temperature rise rate of 5 °C/min. The X-ray diffraction (XRD) profile of the untreated sample was measured by SmartLab (Rigaku Corporation, Akishima, Japan).

The untreated and heat-treated samples were punched at Φ18 mm to make circular samples. All the samples were aligned to 32 sheets of 0.253 g and neatly stacked to form a cylindrical shape. This shape was then used as one set of samples.

### 2.2. Compression Evaluation Method

**Figure 1:**
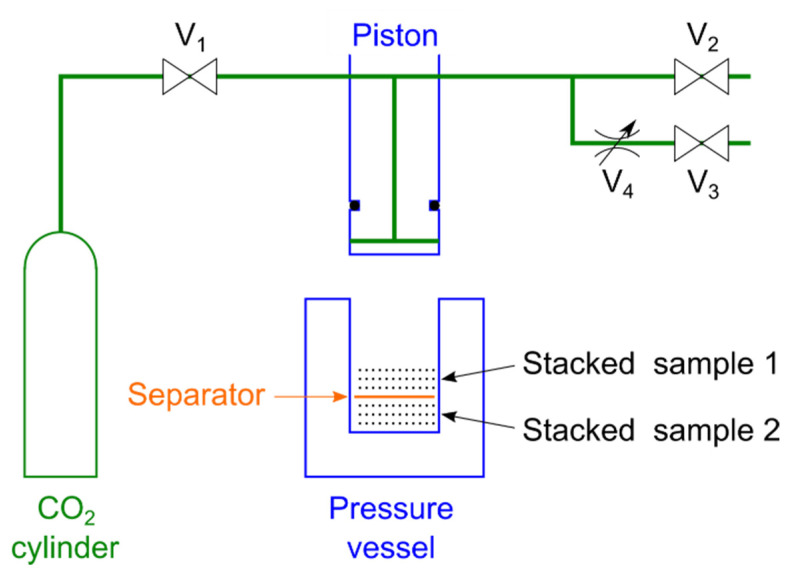
Experimental equipment diagram. The pressure vessel and the piston are sealed with an O-ring on the side of the piston. The samples are put with a separator sandwiched between layers so that the layers do not stick to each other. V_1_: introduction valve; V_2_: high-speed exhaust valve; V_3_: low-speed exhaust valve; V_4_: metering valve.

A schematic diagram of the experimental setup is shown in Figure 1. The sample was prepared in a high-pressure vessel with a separator in the middle of the vessel and samples placed on top of the separator and below the separator. The piston was lowered to a position where the total thickness of the layers of the sample, excluding the separator, was 4 mm. The introduction of CO_2_ and exhaustion of O_2_ were repeated three times while controlling the V_1_ and V_2_ valves to replace the air in the high-pressure vessel with only CO_2_. Afterward, CO_2_ was introduced, and the piston was lowered to the press position. The position where the total thickness of the sample layer was recorded was at a height of 2.0–3.6 mm of the vessel. After holding the piston in the press position for 10 s, the exhaust valve V_3_ was opened and the gas was exhausted for 30 s through the metering valve V_4_, and then the V_2_ valve was opened to release the air. The samples were removed by raising the piston, and the center thickness of each sample was measured with a micrometer. This experiment was conducted at room temperature of 22 °C and the CO_2_ was introduced at a pressure of 6 MPa. The experiment was performed twice with one sample on top of the separator and twice with the same sample below the separator. The average of these four experiments was used. The surfaces of some samples were observed with a scanning electron microscope (SEM) TM-1000 (Hitachi High-Tech Co., Minato-ku, Japan).

### 2.3. Compression Calculation

The model for compression analysis used in the calculations was constructed when designing the multilayer filter [16]. The following relationship is established between the stress *σ*, Young’s modulus *E*, and strain *ε* of a material.

(1) $$\sigma =E\left(p\right)\varepsilon .$$

**Figure 2:**
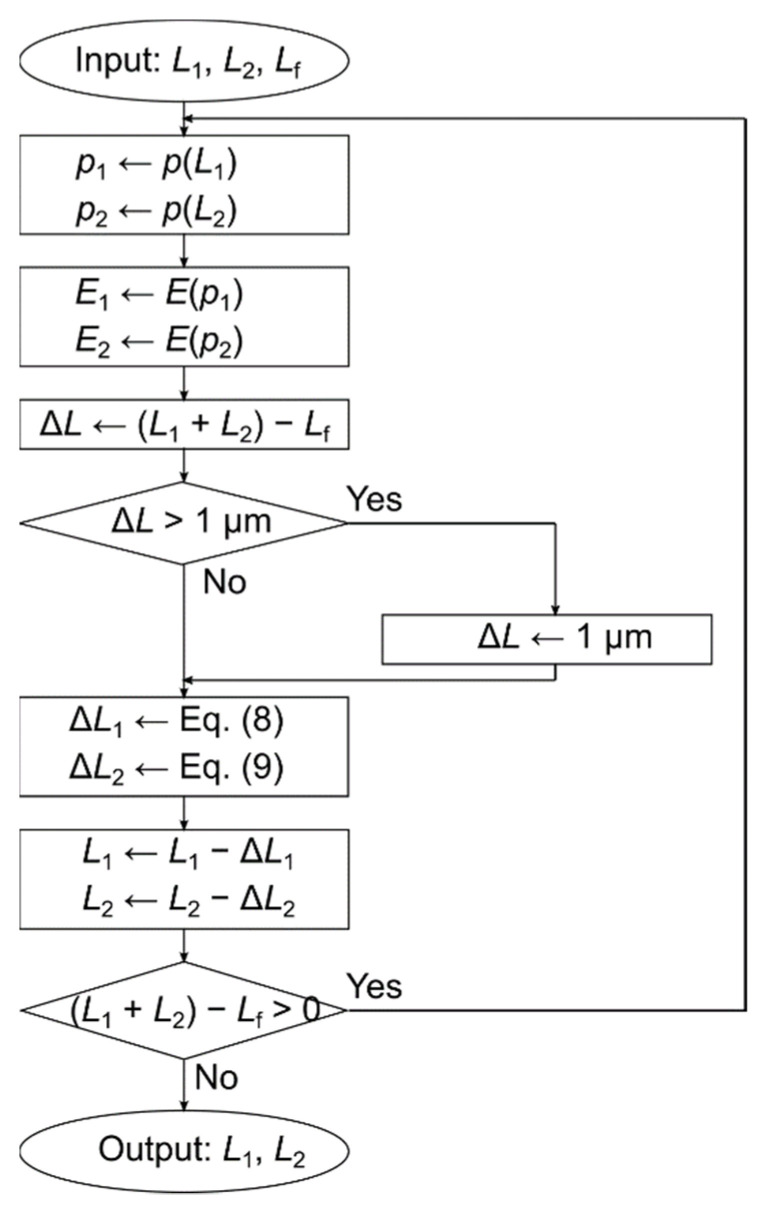
Flowchart of calculating compression. Inputs are *L*_1_: thickness before compression of layer 1, *L*_2_: thickness before compression of layer 2, and *L*_f_: total thickness after compression. Output is *L*_1_: thickness of compression of layer 1 and *L*_2_: thickness of layer 2 after compression. Function *p* is used to find the porosity from the thickness. The porosity is derived by calculating the bulk density from the thickness and then applying Equation (10). Function *E* is used to obtain Young’s modulus from porosity.

Young’s modulus is a function of porosity *p*. When two layers are present, as shown in Stacked sample 1 and Stacked sample 2 in Figure 2, the stress in each layer is demonstrated by the following equations:

(2) $$\sigma_{1}=E_{1}\left(p_{1}\right)\varepsilon_{1}$$

(3) $$\sigma_{2}=E_{2}\left(p_{2}\right)\varepsilon_{2},$$

where the subscripts 1 and 2 indicate layer 1 and layer 2, respectively. In the case of a two-layer sample stacked in a cylindrical shape, if the cross-sections are the same and the stresses (*σ*_1_ and *σ*_2_) are equal, the following relationship is established between Young’s modulus and the strain of each layer.

(4) $$E_{1}\left(p\right)\varepsilon_{1}=E_{2}\left(p\right)\varepsilon_{2}.$$

When the two layers are combined and compressed with total compression thickness, Δ*L*, the following relationship is established between the strains in each layer and their sum.

(5) $$\varepsilon_{1}=\frac{\Delta L_{1}}{L_{1}}$$

(6) $$\varepsilon_{2}=\frac{\Delta L_{2}}{L_{2}}$$

(7) $$\Delta L=\Delta L_{1}+\Delta L_{2},$$

where *L*_1_ and *L*_2_ are the thicknesses of layer 1 and layer 2, respectively. Solving Equations (4)–(7), the amount of compression for each layer is as follows:

(8) $$\Delta L_{1}=\Delta L\frac{L_{1}}{E_{1}\left(p_{1}\right)}\frac{1}{\frac{L_{1}}{E_{1}\left(p_{1}\right)}+\frac{L_{2}}{E_{2}\left(p_{2}\right)}}$$

(9) $$\Delta L_{2}=\Delta L\frac{L_{2}}{E_{2}\left(p_{2}\right)}\frac{1}{\frac{L_{1}}{E_{1}\left(p_{1}\right)}+\frac{L_{2}}{E_{2}\left(p_{2}\right)}}.\;$$

In the case of porous materials, Young’s modulus is known to have porosity dependence. Given that porosity changes as the material is compressed, the above equations are not valid when the compression amount is large. However, the above equations are adaptable when the strain is so small that the dependence on porosity becomes negligible. For this reason, in the previous report, the compression in the calculation was done in 1 µm steps, and the final compression state was calculated by repeating the micro-compression until a given thickness was achieved while updating Young’s modulus. Given that the outer diameter (Φ18 mm) and weight of the sample (0.253 g) were known, the bulk density *ρ*_porous_ of the cylindrical porous material could be easily calculated from the thickness. The difference between *ρ*_porous_ and *ρ*_solid_, the density of the nonporous material (1.34 g/mL), is the pore, and the porosity *p* of the material can be calculated by dividing this difference by *ρ*_solid_, which is shown in the following equation:

(10) $$p=\frac{\rho_{solid}-\rho_{porous}}{\rho_{solid}}$$

As the samples used in this study were those with slightly different crystallinity using the same polymer material, the solid densities of the used models could be considered identical, with an adequate precision of 1.34 g/mL.

The flowchart of the calculation is shown in Figure 2. The input parameters are the initial thickness of the first layer *L*_1_, the initial thickness of the second layer *L*_2_, and the final thickness *L*_f_, while the output parameters are the thickness of the first layer after compression *L*_1_ and the final thickness of the second layer *L*_2_. From the flowchart, the sum of the outputs *L*_1_ and *L*_2_ is equal to the input *L*_f_.

However, this analysis was limited by the fact that the distribution ratio of compression between the two layers is determined by the ratio of Young’s modulus between them, so the absolute values of Young’s modulus of each of the two layers were not determined. Therefore, only the ratio of Young’s modulus was determined, which is a relative index.

### 2.4. Determination of Crystallinity

The crystallinity of the samples was determined by DSC. The degree of crystallinity from the DSC profile is determined by the following equation [28]:

(11) $$Degree\;of\;crystallinity\;\left[\%\right]=\frac{\Delta H_{c}^{1}+\Delta H_{c}^{2}+\Delta H_{m}}{\Delta H_{0}}\times 100\%,$$

where Δ*H*_c_^1^ and Δ*H*_c_^2^ are the enthalpies of cold crystallization and Δ*H*_0_ is the fusion enthalpy of a fully crystallized sample, which has been reported to be 140 J/g for PET [29]. Research has been conducted to apply this formula to PET [30]. In the present system, Δ*H*_c_^2^ and Δ*H*_m_ overlap; however, the value Δ*H*_c_^2^ + Δ*H*_m_ can be determined by taking the integral of the overlapped state. If Δ*H*_c_^2^ + Δ*H*_m_ is denoted by $\Delta H_{c+m}^{2}$, subsequently, Equation (11) becomes

(12) $$Degree\;of\;crystallinity\;\left[\%\right]=\frac{\Delta H_{c}^{1}+\Delta H_{c+m}^{2}}{\Delta H_{0}}\times 100\%.\;$$

## 3. Results and Discussion

**Figure 3:**
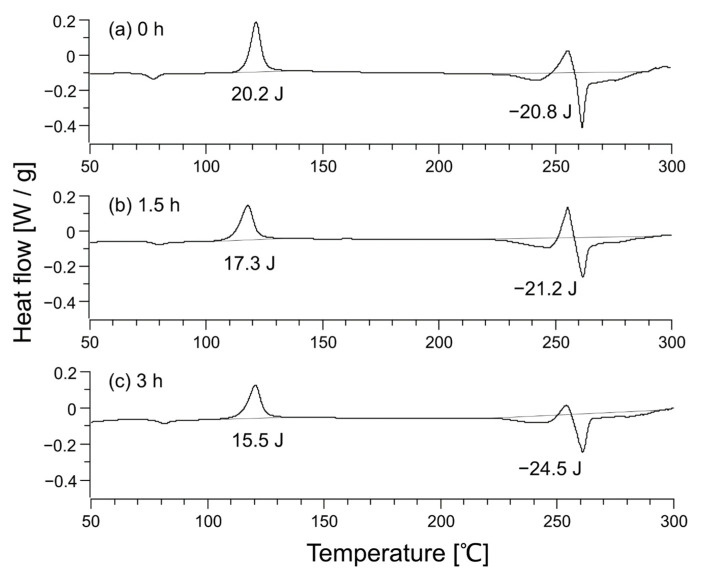
Differential scanning calorimetry profile of unheated sample (**a**), sample after heat treatment for 1.5 h (**b**), and sample after heat treatment for 3 h (**c**). The heat flow in the chart is positive for an exothermic reaction, so the enthalpies listed in the figure correspond to $-\Delta H_{c}^{1}$ and $-\Delta H_{c+m}^{2}$ in Equation (12).

The DSC profiles of the nonwoven fabric before heat treatment, after heat treatment for 1.5 h, and after heat treatment for 3 h are shown in Figure 3. Two peaks of cold crystallization were observed around 125 °C and 250 °C, and the peak of fusion was observed around 260 °C. The position of the cold crystallization peak varied depending on the state of the amorphous part. The case of two cold crystallization peaks was observed in other systems as well [31].

**Figure 4:**
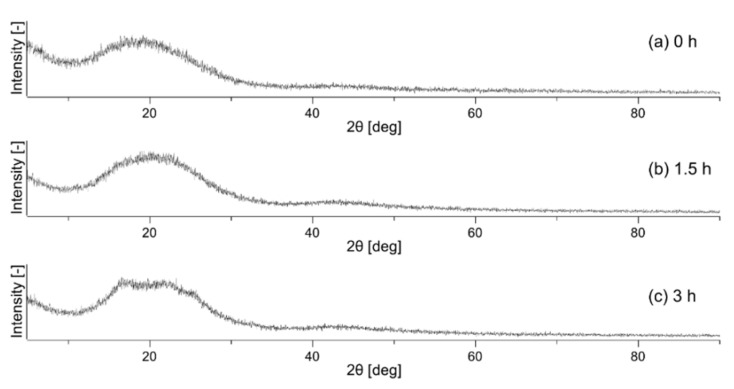
X-ray diffraction profile of unheated sample (**a**), sample after heat treatment for 1.5 h (**b**), and sample after heat treatment for 3 h (**c**).

Using Equation (12), the crystallinity of each sample was calculated to be 0.4% for the untreated nonwoven fabric, 2.8% for the nonwoven fabric heat-treated for 1.5 h, and 6.4% for the nonwoven fabric heat-treated for 3 h. The XRD profiles of nonwoven fabrics are shown in Figure 4. The XRD profile of the polymer shows a broad halo for the amorphous part and a sharp peak for the crystalline part [32]. However, no sharp peaks were observed in Figure 4a, indicating that the crystallinity was close to zero, which is consistent with the DSC result of 0.4% crystallinity for the untreated nonwoven fabric. Figure 4c depicts small peaks overlapping the broad halo, showing a slight crystallization, consistent with the 6.4% crystallinity determined from DSC.

**Figure 5:**
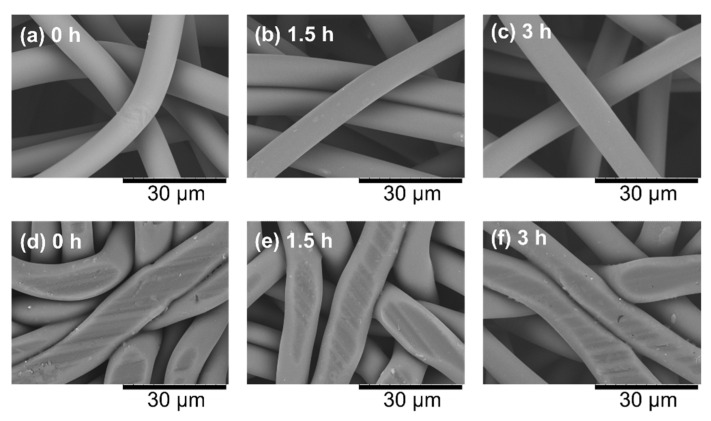
Scanning electron microscope images of the surface of raw nonwoven fabrics and the sample compressed to 1 mm. (**a**) Raw nonwoven fabric without heat treatment, (**b**) raw nonwoven fabric after 1.5 h heat treatment, (**c**) raw nonwoven fabric after 3 h heat treatment, (**d**) CO_2_-assisted polymer compression (CAPC) product made from untreated nonwoven fabric, (**e**) CAPC product using nonwoven fabric heated at 94 °C for 1.5 h as raw material, and (**f**) CAPC product using nonwoven fabric heated at 94 °C for 3 h as raw material.

SEM images of raw nonwoven fabrics are shown in Figure 5a–c. The observation results of the fiber surface when only one type of sample was placed, CO_2_ was introduced at 2 mm, and the sample was compressed to 1 mm are shown in Figure 5d–f. No foaming was observed on the fiber surface because supercritical conditions were not used, the time of exposure to CO_2_ was short, and the fiber diameter was small enough such that the dissolved CO_2_ could easily exit from the fiber. The CO_2_ emitted from the fibers was exhausted through the void between the fibers, so the resulting porous material had through holes. Traces of crushing by the piston were observed on the surface of the fibers, and the press marks on the surface became slightly larger in the order of 3 h treatment, 1.5 h treatment, and untreated, suggesting that the fibers were more crushed. However, the SEM images did not show a clear enough difference to identify the hardness of the fibers.

The results of the two-layer compression test are shown in Table 1. The experiments were conducted under 15 different experimental conditions: A1–A5, B1–B5, and C1–C5. Experiment A1 was conducted with a combination of non-heat-treated nonwoven fabric (heat treatment time: 0 h) and 1.5 h heat-treated nonwoven fabric. When compressed with a piston from the 4 mm CO_2_ introduction position, the layer of non-heat-treated nonwoven fabric was compressed to 1.774 mm, whereas the layer of 1.5 h heat-treated nonwoven fabric was compressed to 1.825 mm. Similarly, Experiment A2 was conducted with the combination of non-heat-treated nonwoven fabric (heat treatment time: 0 h) and 1.5 h heat-treated nonwoven fabric. When compressed with a piston from the 4 mm CO_2_ introduction position, the 0 h nonwoven fabric layer was compressed to 1.557 mm, whereas the 1.5 h heat-treated nonwoven fabric layer was compressed to 1.645 mm. As written in the experiment section, two experiments with one of the two types of samples on top and two experiments with the same type of samples on the bottom, making it a total of four experiments, were conducted. The mean values and standard deviations are shown in Table 1. No difference was recorded between the experiments with the sample on top and those with the sample on the bottom, and there was no dependence of the experiment on the position of the sample. In all experiments, the untreated samples were more compacted than the 1.5 h heat-treated samples, and the 1.5 h heat-treated samples were more compacted than the 3 h heat-treated samples, indicating that the hardness increased in the order of untreated, 1.5 h heat-treated, and 3 h heat-treated samples. This increasing order of hardness of the samples is consistent with the order of crystallinity, suggesting that the degree of plasticization decreases in the order of untreated, 1.5 h heat-treated, and 3 h heat-treated samples. This order of the degree of plasticization is consistent with those of amorphous area, showing that plasticization is caused by the impregnation into the amorphous part of the polymer.

Model calculation was performed to derive the relationship between the porosity and Young’s modulus of the porous material in the presence of CO_2_. Although the initial thickness of the porous material is necessary for the model calculation, the thickness of each layer in the initial state when each layer of the nonwoven fabric is placed in a stack and compressed by a piston could not be ascertained because the stainless steel container was opaque. Therefore, the following considerations were obtained. The position of CO_2_ introduction was the same regardless of the experimental conditions, so for example, Experiment A2 was considered to have been reached after going through the state of Experiment A1 during the compression process by the piston. Similarly, Experiment A3 was considered to have been reached after going through the state of Experiment A1 and that of Experiment A2. When the state before the final state was known, the result of Experiment A1 could be used as the initial value for the calculation performed to compress the final state to the total value of each layer of Experiment A2, and then to check how well the result agreed with the experimental result. Similarly, for Experiment A3, the result of Experiment A2 was used as the initial value for the calculation performed to compress the final state to the total value of each layer of Experiment A3. This operation was performed for each experiment, and fitting was performed so that the least-squares error between the experimental and calculated values would be small. The parameters were determined using the Nelder–Mead method [33], which does not require derivation of derivatives for fitting. In incorporating the Nelder–Mead method into the program, Reference [34] was employed. The fitting of the results was performed on only 12 out of the 15 datasets (A2–A5, B2–B5, C2–C5) because the model calculation used the previous data to establish the initial value. The number of fitting parameters varied from two to four, depending on the equation to be fitted, and the amount of fitting data required was sufficiently larger than the amount of fitting parameters.

The Young’s modulus of the CAPC product was calculated by conversion from hardness measurement [35]. Several equations have been proposed for the elastic modulus of porous materials, such as ceramics [36], metals [37], and unspecified materials [38]. These equations are largely classified into those with and without critical porosity. However, in a previous paper [35], the following simple equation without critical porosity was presented:

(13) $$E\left(p\right)=E_{0}\cdot {\left(1-p\right)}^{f},\;$$

where Young’s modulus *E*_0_ of the solid without pore was 266 MPa and the shape factor *f* was 2.41. Equation (13) is for the case when CO_2_ is removed after CAPC and the material returns to its unplasticized state. Assuming that the shape of the curve is unaltered in the presence of CO_2_ and only the absolute value changes, Young’s modulus as a function of porosity was assumed to be the following equations:

(14) $$E_{0h}\left(p\right)={\left(1-p\right)}^{2.41}.\;$$

(15) $$E_{1.5h}\left(p\right)=C_{1.5h}\cdot {\left(1-p\right)}^{2.41}.\;$$

(16) $$E_{3h}\left(p\right)=C_{3h}\cdot {\left(1-p\right)}^{2.41}.\;$$

**Figure 6:**
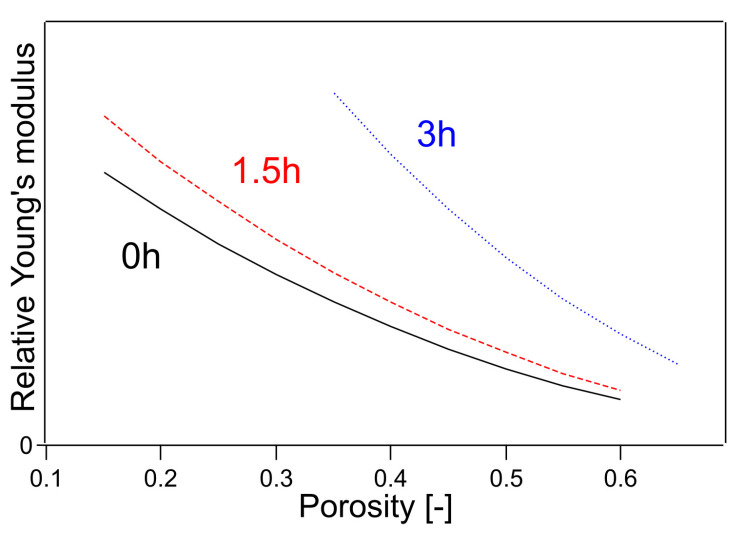
Young’s modulus fitted by Equations (14)–(16). The numbers in the figure indicate the heating time of the sample.

The parameters *C*_1.5h_ and *C*_3h_ were determined using the least-squares method. In Equation (14), Young’s modulus is 1 when the porosity of the untreated sample is 0. This outcome is because this method can only determine the relative value of Young’s modulus. The Young’s modulus determined by this method is shown in Figure 6. In Figure 6, Young’s modulus is plotted for a limited porosity range because Young’s modulus is plotted for the porosity only in the fitting range. The results of the calculations are shown in Table 1, where the value of the coefficient of determination R^2^ was 0.9928.

**Figure 7:**
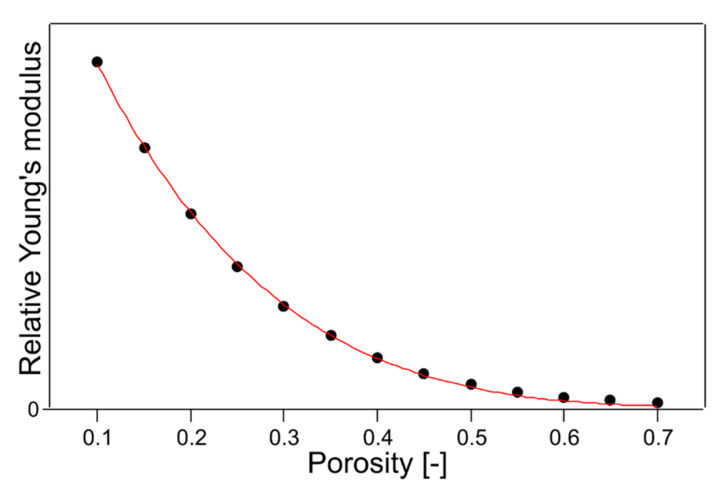
The plot of the quintic function used when designing the multilayer filter [16] and the fitting result with Equation (13).

As in the previous study [16], which fabricated a multilayer filter using the same untreated nonwoven fabric used in this study, Young’s modulus was determined to simulate the thickness of each layer. In addition, a fifth-order polynomial was used to fit the results. These results, fitted by the fifth-order polynomial and the result of fitting with Equation (13), are shown in Figure 7. These results agree extremely well with the general porosity and Young’s modulus equations, and the fitting parameter *f* was 4.77 at that time. Therefore, the following equations using this parameter *f* = 4.77 were used as the next trial.

(17) $$E_{0h}\left(p\right)={\left(1-p\right)}^{4.77}.\;$$

(18) $$E_{1.5h}\left(p\right)=C_{1.5h}\cdot {\left(1-p\right)}^{4.77}.\;$$

(19) $$E_{3h}\left(p\right)=C_{3h}\cdot {\left(1-p\right)}^{4.77}.\;$$

**Figure 8:**
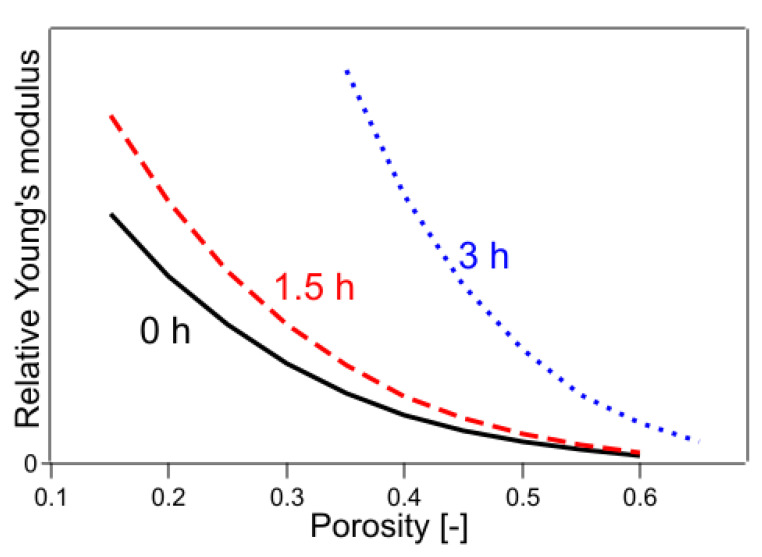
Young’s modulus fitted by Equations (17)–(19). The numbers in the figure indicate the heating time of the sample.

The results are shown in Table 1 and Figure 8. The value of R^2^ was 0.9944, which was better than the previous result using Equations (14)–(16), indicating that Young’s modulus trend in the presence of CO_2_ was still slightly different from that in the absence of CO_2_.

Here, a question arose as to whether the functional form of Young’s modulus in the presence of CO_2_ would be the same regardless of the degree of crystallinity. The previously reported functional form was for the untreated sample. Therefore, Young’s modulus was calculated using the following equations:

(20) $$E_{0h}\left(p\right)={\left(1-p\right)}^{4.77}$$

(21) $$E_{1.5h}\left(p\right)=C_{1.5h}\cdot {\left(1-p\right)}^{f_{1.5h}}$$

(22) $$E_{3h}\left(p\right)=C_{3h}\cdot {\left(1-p\right)}^{f_{3h}}$$

**Figure 9:**
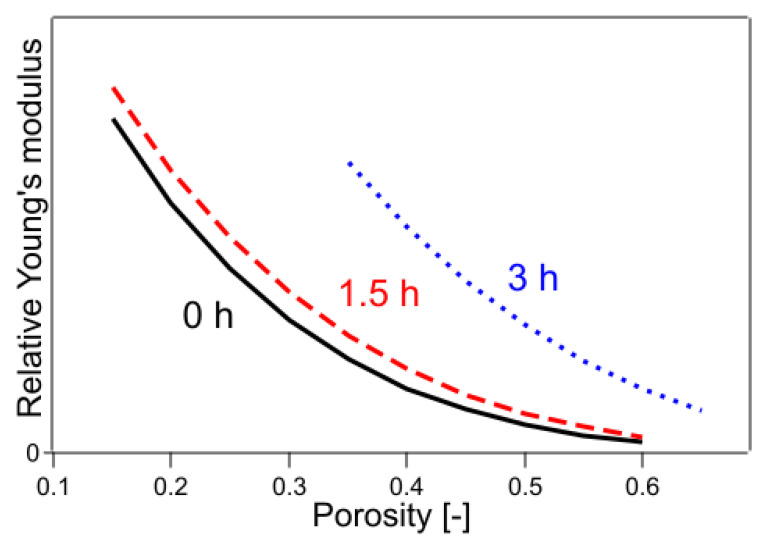
Young’s modulus fitted by Equations (20)–(22). The numbers in the figure indicate the heating time of the sample.

In Equations (20)–(22), the exponential part of the function was fixed only for the untreated sample, whereas the exponential part of each of the remaining samples was used as the fitting parameter. The results are shown in Table 1 and Figure 9. The value of R^2^ was 0.9987, which was a significant improvement. The results of the fitting parameters were as follows: *C*_1.5h_ was 1.01, *C*_3h_ was 1.55, *f*_1.5h_ was 4.26, and *f*_3h_ was 3.14. *C*_1.5h_ and *C*_3h_ represent the Young’s modulus of a solid without pores, showing that the Young’s modulus of a sample with 2.8% crystallinity was 1.01 and that with 6.4% crystallinity was 1.55, when the crystallinity of the sample with 0.4% crystallinity was 1. The *f*_1.5h_ and *f*_3h_ are shape factors. In the case of the present sample, because the fibers were stacked horizontally and compressed, the elastic modulus was considered a mixture of tensile and flexural modulus. The contribution of flexural modulus was expected to be larger on the low-density sample, and the contribution of tensile modulus was expected to be larger as it approached high-density pore-free solids. It is well known that the flexural modulus is generally lower in value than the tensile modulus of elasticity for practical purposes [39]. In the present case, the effect of mixing the tensile and flexural modulus was included in the shape factor, and the shape factor was regarded to have changed with crystallinity. As the degree of crystallinity increased, the functional form approached that of Young’s modulus of the CAPC porous material (*f* = 2.41) after CAPC treatment. Figure 9 shows that Young’s modulus increases in the order of untreated, 1.5 h heat-treated, and 3 h heat-treated samples, indicating the increasing hardness of the material in that order. In particular, comparing Young’s modulus of untreated and 3 h heat-treated specimens, Young’s modulus of 3 h heat-treated specimens with a porosity of 0.4 was about four times higher than that of untreated specimens, and Young’s modulus of 3 h heat-treated specimens with a porosity of 0.5 was about five times higher than that of untreated specimens, indicating a large difference.

The present results show that Young’s modulus of CAPC porous materials in the presence of CO_2_ can be expressed in a very simple description without critical porosity, which can be used to describe the experimental results. It has also been shown that Young’s modulus changed significantly even after heat treatment for 3 h, although the degree of crystallinity at that time was as low as 6.4%. Considering that the categorization of the CAPC method is crimping, which is highly dependent on the hardness of the material selected, it is important to keep the crystallinity of the raw fiber very low and to use a material with a low Young’s modulus in the presence of CO_2_.

## 4. Conclusions

By heat treating the raw material fibers at low temperatures for a given period, samples with different degrees of crystallinity were prepared without changing the fiber shape. These samples were subjected to CAPC treatment in two overlapping layers, and the difference in compressibility of each layer was measured. The measured results were simulated using the analysis of strain by Young’s modulus. The relative magnitude of Young’s modulus of the samples with different crystallinity values and its functional form with respect to porosity were studied. It was shown that Young’s modulus of the porous polymer in the presence of CO_2_ changed significantly with a small difference in crystallinity, and that with increasing crystallinity, Young’s modulus approached the functional form in the atmosphere without CO_2_. Given that the degree of crystallinity of the nonwoven fabric made by the melt blowing method was extremely low, it can be inferred that the CAPC method is more effective if the nonwoven fabric in its state of low crystallinity after melt blowing is used as the raw fiber in its original state.

## Figures and Tables

**Table 1:** Experimental results and model calculation results.

Experiment Name	Heat Treatment Time [h]	Thickness [mm]	Fitting Results of Equations (14)–(16) [mm]	Fitting Results of Equations (17)–(19) [mm]	Fitting Results of Equations (20)–(22) [mm]
A1	0	1.774 ± 0.007			
	1.5	1.825 ± 0.008			
A2	0	1.557 ± 0.012	1.569	1.564	1.558
	1.5	1.645 ± 0.014	1.634	1.638	1.645
A3	0	1.355 ± 0.012	1.357	1.357	1.354
	1.5	1.446 ± 0.012	1.444	1.444	1.447
A4	0	1.159 ± 0.009	1.158	1.159	1.161
	1.5	1.241 ± 0.010	1.241	1.241	1.239
A5	0	0.971 ± 0.002	0.964	0.966	0.973
	1.5	1.030 ± 0.004	1.036	1.035	1.028
B1	0	1.523 ± 0.011			
	3	2.076 ± 0.012			
B2	0	1.327 ± 0.008	1.342	1.337	1.317
	3	1.875 ± 0.005	1.860	1.865	1.885
B3	0	1.138 ± 0.015	1.155	1.155	1.148
	3	1.662 ± 0.016	1.646	1.646	1.653
B4	0	0.983 ± 0.018	0.976	0.980	0.987
	3	1.417 ± 0.018	1.424	1.420	1.412
B5	0	0.855 ± 0.018	0.818	0.821	0.845
	3	1.147 ± 0.018	1.184	1.181	1.156
C1	1.5	1.579 ± 0.028			
	3	2.021 ± 0.027			
C2	1.5	1.381 ± 0.020	1.395	1.389	1.374
	3	1.821 ± 0.019	1.807	1.813	1.828
C3	1.5	1.190 ± 0.009	1.204	1.202	1.196
	3	1.612 ± 0.009	1.597	1.599	1.605
C4	1.5	1.015 ± 0.013	1.019	1.021	1.024
	3	1.383 ± 0.014	1.378	1.376	1.373
C5	1.5	0.877 ± 0.013	0.849	0.851	0.866
	3	1.124 ± 0.008	1.152	1.150	1.135

The thickness error is the standard deviation obtained from four experiments. The total value of each layer in the calculation result may not match the experimental result due to rounding error.

## Data Availability

All data generated or analyzed during this study are included in this published article.
